# The effects of acute exercise and inflammation on immune function in early-stage prostate cancer

**DOI:** 10.1016/j.bbih.2022.100508

**Published:** 2022-09-07

**Authors:** Tim Schauer, Sissal Sigmundsdóttir Djurhuus, Casper Simonsen, Klaus Brasso, Jesper Frank Christensen

**Affiliations:** a Centre for Physical Activity Research, Copenhagen University Hospital, Copenhagen, Denmark; b Copenhagen Prostate Cancer Center, Department of Urology, Copenhagen University Hospital, Copenhagen, Denmark; c Department of Clinical Medicine, University of Copenhagen, Denmark; d Institute of Exercise and Biomechanics, University of Southern Denmark, Denmark; e Digestive Disease Center, Bispebjerg Hospital, Copenhagen, Denmark

**Keywords:** Natural killer cells, T-lymphocytes, Exercise, Prostate cancer, Inflammation, PCa, Prostate Cancer, NK, Natural killer, NKCA, Natural killer cell cytotoxic activity, PBMCs, Peripheral Blood Mononuclear Cells, PSA, Prostate-Specific-Antigen, MFI, Mean Fluorescence Intensity, EMD + 95% CI, Estimated Mean Difference with 95% Confidence Interval

## Abstract

**Background:**

The immune system plays a vital role in cancer development and progression. Strategies mobilizing cytotoxic cells of the immune system to combat immunosuppression in cancer may help to improve the treatment response of patients. To this end, we aimed to characterize the anti-cancer effect of acute exercise, including the involvement of inflammatory signals.

**Patients and methods:**

Twenty patients with early-stage prostate cancer (PCa) scheduled to undergo prostatectomy performed one bout of acute exercise consisting of a watt-max test and four high-intensity intervals. Natural Killer (NK), NKT-like and T cell phenotype, NK cell cytotoxic activity (NKCA), and NKCA per-cell against cell lines of leukemia (K562) and prostate cancer origin (LNCaP and PC-3) were assessed. Inflammatory markers (TNF-α, IL-6, and CRP) were measured in plasma.

**Results:**

Exercise increased NK, NKT-like, and CD8 T cell concentration in the circulation. Furthermore, exercise shifted immune cells towards a mature and cytotoxic phenotype e.g., NK cells exhibited higher CD57 as well as lower NKG2A expression. NKT-like and CD8 cells exhibited elevated CD57, TIGIT and Granzyme-B expression. Exercise significantly improved NKCA against K562 (+16% [5%; 27%]; p = 0.002) and LNCaP (+24% [14%; 34%]; p < 0.001) but not PC-3. NKCA per NK cell decreased during exercise and increased 1-h post exercise compared to baseline in K562, LNCap, and PC-3 cell lines. Baseline IL-6 correlated with lymphocyte, monocyte and T cell concentration pre-exercise and inversely correlated with the fold-change of mobilized lymphocytes and CD8 T cells during exercise. Furthermore, baseline IL-6 and TNF-α inversely correlated with NKCA against PC-3 cells during exercise.

**Conclusions:**

Acute exercise mobilized cytotoxic immune cells and improved NKCA in patients with PCa whereas low-grade inflammation might impair the response. Whether the observed improvements impact long-term outcomes warrant further investigation.

**Clinical trial number:**

NCT03675529.

## Highlights

• Exercise mobilizes NK, CD8 T and NKT-like cells in patients with prostate cancer.
• Immune cells exhibit mature and cytotoxic phenotype during high-intensity exercise.
• NK cytotoxic activity against K562 and LNCaP but not PC3 increases with exercise.
• Markers of inflammation inversely correlate with exercise-enhanced immune function.

## 1 Introduction

The immune system plays an important role in combating cancer, as cytotoxic immune cells e.g., NK and T cells, constitute the “first line” of defense against carcinogenic cellular events (Strasner and Karin, 2015). Suppression of these cytotoxic immune cells e.g., by an immunosuppressive environment, is a key contributing factor in cancer development and progression (Stultz and Fong, 2021). Hence, conditions promoting immune cell suppression such as obesity are linked to cancer risk (Nathan, 2008; Lavalette et al., 2018), possibly through accompanying chronic low-grade inflammation which can impair NK and T cell function (Aguilar and Murphy, 2018; Bähr et al., 2020). Moreover, tumor-derived inflammatory cytokines, elevated in patients with active disease, are linked to NK cell impairment (Wu et al., 2019). Accordingly, strategies to prevent or control immune suppression are emerging as potential adjuncts to conventional treatment regimens (Stultz and Fong, 2021).

Physical exercise training comprises a relatively low-risk and low-cost strategy with significant immunomodulatory capacity which may hold important anti-cancer potential (Hojman et al., 2017). A rapid release and redistribution of all major immune cell subsets occurs in response to acute exercise (Campbell and Turner, 2018; Pedersen and Hoffman-Goetz, 2000). Intriguingly, early pre-clinical data suggest that exercise-induced immune cell mobilization may comprise a direct tumor-growth inhibiting mechanism (Pedersen et al., 2016). In healthy individuals, acute exercise mobilizes mature and cytotoxic CD56^dim^ NK and CD8^+^ T cells in an intensity-dependent manner and improves NK cell cytotoxic activity (NKCA) against certain cancer cell lines (Pedersen and Hoffman-Goetz, 2000; Campbell et al., 2009; Simpson et al., 2007, 2008; Zimmer et al., 2017; Rumpf et al., 2021), thus indicating strengthened anti-cancer immunity during and potentially also following exercise.

Yet, despite these promising reports, several gaps remain to be elucidated to uncover the full potential of exercise as a strategy to increase the anti-cancer capacity of the immune system. First, the effect of acute exercise on anti-cancer immunity has largely been studied in only healthy individuals (Zimmer et al., 2017), who may differ significantly in both exercise capacity/response as well as their (patho-) physiological profile compared to patients with cancer. Second, the current focus centers on the anti-cancer effects of acute exercise-conditioned serum (Dethlefsen et al., 2016; Metcalfe et al., 2021), with little attention to the impact of the (accompanying) systemic cell composition. Lastly, studies with emphasis on immune cells investigated exercise-induced changes in cell concentrations with little attention to the possible role of exercise-induced changes in immune cell function i.e., NKCA and immune cell phenotype.

Against this background, we performed a comprehensive evaluation of the effect of one single bout of aerobic exercise on immune function in patients with cancer before the onset of any anti-cancer therapy. We utilized an experimental setup, where newly diagnosed patients with early-stage prostate cancer (PCa) performed a single high-intensity exercise bout the day prior to radical prostatectomy. We explored systemic changes in NK and T cell mobilization and phenotype and the corresponding changes in NKCA against PCa cell lines. As an explorative evaluation, we investigated associations between baseline systemic inflammation and the effect of acute exercise on immune function.

## 2 Methods

### 2.1 Participants

This secondary analysis includes 20 patients with histologically verified localized prostate adenocarcinoma scheduled to undergo curative intended radical prostatectomy, included between October 2018 and November 2019 (Clinical Trials identifier: NCT03675529) (Djurhuus et al., 2022a). Exclusion criteria: age <18 years, other malignancy requiring active treatment, Eastern Cooperative Oncology Group (ECOG) or World Health Organization (WHO) performance status >1, current treatment with beta-blockers, physical disabilities contradicting exercise, allergy to pimonidazole or the inability to read and understand Danish (Supplementary Fig. 1A). Comorbidities were assessed during the telephone screening. Sedentary behavior was assessed using the International Physical Activity Questionnaire (IPAQ) - Short Form. Gleason score and days since diagnosis were extracted from medical records. All study participants gave informed consent before initiating the study and the work was carried out in accordance with the Declaration of Helsinki.

### 2.2 Acute exercise test

The present study describes a mechanistic study investigating the effect of one acute bout of exercise on immune-related parameters the day before radical prostatectomy (Supplementary Fig. 1B). The exercise bout consisted of a watt-max test followed by four high-intensity intervals. Patients performed the watt-max test as a maximal incremental exercise test on an electronically braked bicycle ergometer (LC4, Monark, Varberg, Sweden). Patients initiated the test with a 3 min warm-up at 70 watts followed by an incremental increase of 20 watts every minute until exhaustion. Watt-max calculation: last completed workload (watt) + 20/60 * number of seconds in the last interval. Estimated VO_2_max [ml O_2_/min] was calculated as 10.8 * watt-max/weight [kg] + 7 (Swain, 2000). Immediately after the watt-max test, patients performed 10 min of light pedaling at 30% of watt-max, followed by four 1-min high-intensity intervals at 100% of watt-max, interspersed by 3 min of recovery at 30% of watt-max (Supplementary Fig. 1B).

### 2.3 Blood samples

Fasting blood samples from the antecubital vein were obtained at resting pre-exercise (baseline) conditions, immediately after the watt-max test, immediately (within 1 min) after the last high-intensity interval, and after 1 h of rest (1h post-EX) (Supplementary Fig. 1B). For plasma isolation, blood was collected in EDTA tubes, centrifuged at 2000*g and 4 °C for 10 min, aliquoted and stored at 4 °C until long-term storage at −80 °C within 2 h of isolation. We assessed CRP, IL-6, and TNF-α with R-plex Human CRP kit and V-plex pro-inflammatory panel 1 (MesoScaleDiscovery, USA, Maryland) according to the manufacturer's guidelines using electrochemiluminescence. In brief, plasma samples were added to 96-well plates precoated with specific antibodies. After overnight incubation at 4 °C, plates were washed using an automated plate washer and secondary antibodies were added for 2 h while shaking. Finally, plates were washed again and read buffer was added and the plate was measured on a Quickplex-120 (MesoScaleDiscovery). Prostate-Specific-Antigen (PSA) was measured at the Department of Clinical Biochemistry, Rigshospitalet, Denmark, using a sandwich electrochemiluminescence-immunoassay (Cobas 8000, Roche Diagnostics, Switzerland). All analyses regarding blood samples were performed blinded.

### 2.4 Flow cytometry

Absolute differential cell counts of leucocytes i.e., lymphocytes, neutrophils, and monocytes were obtained from the Department of Clinical Biochemistry at Rigshospitalet, Denmark, based on automated flow cytometric assessment of forward and side scatter (Sysmex XN, Kobe, Japan). Absolute cell counts of lymphocyte subsets i.e., NK (CD3^−^ CD14^−^ CD16^+^ CD56^dim^ and CD3^−^ CD14^−^ CD16^+/-^ CD56^bright^), NKT-like (CD3^+^ CD56^+^), CD8 T cells (CD3^+^ CD8^+^ CD56^−^) and total T cells (CD3^+^) were calculated based on proportions [%] in lymphocytes (Supplementary Fig. 2), obtained in separately performed flow cytometry experiments in combination with the absolute number of lymphocytes from the differential counts of leucocytes.

Blood samples used for the determination of lymphocyte subpopulations were stored at room temperature until the exercise bout was finished. Next, all blood samples were processed simultaneously to isolate peripheral blood mononuclear cells (PBMCs) via density centrifugation with lymphoprep solution (Stemcell Technologies, Canada) and leucosep tubes (Greiner bio-one, Austria). In brief, blood was layered on top of the membrane and centrifuged for 15 min at 800g without the use of a break. Next, the PBMCs layer was transferred and washed once in FACS buffer (PBS + 2% FBS).

For staining of surface markers, 400.000 PBMCs (Countess 3 FL Automated Cell Counter, ThermoFisher, USA) were incubated with FC-block and live-dead staining solution for 15 min at RT, washed once and stained with antibody cocktails (Supplementary Table 1) for 30 min at 4 °C using brilliant stain buffer (BD Bioscience, USA). Next, PBMCs were washed, fixated in 2% paraformaldehyde for 10 min at RT, washed, and finally resuspended in FACS buffer for acquisition within the next 24 h. Intracellular antibody staining for perforin and granzyme B was performed by resuspending PBMCs in permeabilization buffer containing antibody cocktails followed by 30 min of incubation at 4 °C and a subsequent washing step before storage in FACS buffer until acquisition. Internal controls (cryopreserved PBMCs) to verify staining, fluorescence-minus-one controls to support the gating of true positive signals and single-color stains for compensation were always included. Internal controls revealed a CV below 12% for T, NKT-like, and NK cell proportion [%] of lymphocytes across all measurement days.

PBMCs were acquired using an LSRFortessa equipped with 5 lasers (488 nm, 640 nm, 405 nm, 561 nm, 355 nm) maintained by the flow cytometry core facility at Copenhagen University using FACSDiva software v.8.01 (BD Biosciences, USA). Analyses of samples (gating strategy is available in Supplementary Fig. 2) and compensation calculation for each participant were performed in FlowJo v.10.6.1 (BD Biosciences, USA).

### 2.5 NK cell cytotoxic activity (NKCA) assay

Cancer cell lines K562, LNCaP (clone FGC), and PC-3 were obtained from the European collection of authenticated cell cultures and kept below 30 (K562, PC3) or below 35 (LNCaP) passages. K562 and PC-3 cells cultivated in RPMI1640 + 10% FBS (ThermoFisher, USA), while LNCaP were supplied with additional 1 mM sodium pyruvate (Sigma Aldrich, USA). We implemented the NKCA assay during the study and therefore only a consecutive subset of patients is available (Supplementary Fig. 1A).

We used a calcein-based killing assay to assess NKCA (Neri et al., 2001). Briefly, cancer cells were washed in FBS-free media and incubated in 15 μM calcein-AM (AAT Bioquest, USA) for 30 min at 37 °C, followed by two additional washes. Isolated PBMCs (see above) were stored at room temperature for 2 h and subsequently washed once in FBS-free media. PBMCs and K562 as well as LNCaP cancer cells (cell concentration based on Countess 3 FL Automated Cell Counter, ThermoFisher, USA) were combined in ratios ranging from 50:1 to 5:1 while PC-3 ratios ranged from 25:1 to 10:1 due to sample limitation. Combined cells were incubated for 4 h at 37 °C and 5% CO_2_. Spontaneous calcein release (negative control) and maximal release (positive control; 2% Triton X-100) were monitored. Calcein signal in the supernatant was measured in triplicate wells using a FLUOstar Optima (BMG Labtech, Germany). NKCA [%] calculation: (sample signal – spontaneous release)/(maximal – spontaneous release) * 100 (Neri et al., 2001). For each experiment, 5000 K562, 7500 LNCaP and 15000 PC-3 cells were used as target cells. In initial experiments, target cell numbers were determined by the maximal signal difference between positive and negative control. NKCA per NK-cell calculation: the absolute amount of killed cancer cells was divided by the estimated amount of NK cells (target cells * ratio * NK cell proportion in PBMCs).

### 2.6 Statistical analysis

The effect of exercise on immune function was modelled using linear mixed models. The concentration (or cell marker expression) was included as the dependent variable with timepoint (four levels: baseline/watt-max/intervals/1h post-EX) as a fixed effect. Additionally, the baseline concentration (or expression level) was included as a covariate and a random effect (i.e. random intercept) of participants was added to account for repeated measures. If a main effect of time was found, post-hoc comparisons for baseline vs. watt-max, baseline vs. intervals, and baseline vs. 1h post EX were performed using the Sidak correction for multiple testing. The dependent variable was log-transformed to improve model compliance if not stated otherwise. Therefore, estimated mean differences (EMD) are presented as relative changes with 95% confidence intervals (CI) i.e., an EMD of 1.10 represents a relative increase of 10% from e.g., baseline to the post watt-max test. Model compliance was assessed by residual and quantile plots. Surface marker expression is given as proportion [%]. If the proportion was not available due to insufficient separation of positive and negative cells, mean fluorescence intensity (MFI) values were used. For MFI values, only statistically significant changes with more than 10% difference were interpreted as biologically significant. The spearman correlation coefficient was used to evaluate the relationship between baseline inflammatory variables and NKCA (ratio 50:1) against cancer cell lines. The fold change was calculated e.g., as the post watt-max test divided by baseline - 1. Data is presented as EMD +95% CI if not stated otherwise. A *p-*value below 0.05 was regarded as statistically significant. All analyses were performed using R (v. 3.6.0) via RStudio (v. 1.2.1335) and the package lm4 (v.1.1–26) (Bates et al., 2015) was used for linear mixed models.

## 3 Results

In total, 20 patients with early-stage PCa performed one acute bout of exercise the day before the scheduled prostatectomy (Table 1; Supplementary Fig. 1).

**Table 1:** Patient characteristics.

n	20

Age [years]	64 (53–77)
BMI [kg/m^2^]	26 (20–32)
Body weight [kg]	82 (58–96)
Waist-to-hip ratio	0.99 (0.81–1.11)
Sedentary behavior* [hours]	6 (3–12)
Gleason score**	7 (6–9)
Days since diagnosis	76 ± 31
PSA [ng/ml]	7.8 (2.4–54)
resting HR [beats per minute]	67 ± 10
max HR [beats per minute]	158 ± 16
Workload [watt]	220 ± 52
estimated VO_2_ peak [mlO_2_/kg/min]	37 ± 9
CRP [mg/L]	3.4 ± 4.7
IL-6 [pg/mL]	0.8 ± 0.5
TNF-α [pg/mL]	3.0 ± 1.2

Comorbidities	n (%)

Hypertension	7 (35)
Cardiovascular disease	2 (10)
Hypercholesterolemia	2 (10)
Asthma	1 (5)
Diabetes	1 (5)
Other***	2 (10)

Depicted are resting pre-exercise (baseline) patient characteristics as mean ± SD or mean (range) if not stated otherwise. Exercise-related characteristics are based on the watt-max test. *Hours per average week-day using self-reported data, **Gleason score from prostatectomy, ***other include (n = 1 arthrosis, n = 1 green star). *Abbreviations: BMI (body mass index), PSA (prostate specific antigen), HR (heart rate), CRP (C-reactive protein), IL (interleukin), TNF (tumor necrosis factor).*

### 3.1 The effect of acute exercise on immune cell phenotype

Acute exercise led to a significant increase in monocyte, neutrophil, and overall lymphocyte concentration. During the 1-h recovery period post-exercise, monocytes returned to baseline values while neutrophils remained elevated, and lymphocytes dropped below resting pre-exercise baseline values (Table 2) (Djurhuus et al., 2022a).

**Table 2:** The effect of acute exercise on immune cell concentrations.

	Raw data [Mean ± SD]				Relative change from baseline to					
	Baseline	Watt-max	Intervals	1h post-exercise	Post watt-max test		Post intervals		1h post-exercise	
					EMD (95% CI)	*p*	EMD (95% CI)	*p*	EMD (95% CI)	*p*
Immune cells [10^3^ cells/μl]										
Monocytes	0.5 ± 0.2	0.8 ± 0.3	0.9 ± 0.3	0.6 ± 0.2	1.41 (1.21; 1.64)	***	1.55 (1.32; 1.81)	***	1.03 (0.88; 1.20)	ns
Neutrophils	3.7 ± 0.9	4.9 ± 1.3	5.0 ± 1.2	4.7 ± 1.2	1.33 (1.20; 1.46)	***	1.36 (1.23; 1.50)	***	1.26 (1.14; 1.39)	***
T cells	1.02 ± 0.34	1.72 ± 0.81	1.71 ± 0.81	0.93 ± 0.31	1.64 (1.37; 1.97)	***	1.62 (1.35; 1.94)	***	0.90 (0.75; 1.08)	ns
NKT-like cells	0.12 ± 0.09	0.42 ± 0.41	0.36 ± 0.35	0.07 ± 0.05	3.18 (2.63; 3.85)	***	2.82 (2.33; 3.41)	***	0.63 (0.52; 0.76)	***
CD8 T cells	0.20 ± 0.13	0.48 ± 0.40	0.45 ± 0.38	0.16 ± 0.09	2.32 (1.89; 2.85)	***	2.12 (1.72; 2.61)	***	0.82 (0.66; 1.01)	ns
CD56^dim^ NK cells	0.21 ± 0.09	1.43 ± 0.65	1.06 ± 0.56	0.09 ± 0.06	6.57 (5.28; 8.19)	***	4.68 (3.76; 5.83)	***	0.40 (0.32; 0.50)	***
CD56^brigh^^t^ NK cells	0.01 ± 0.01	0.03 ± 0.01	0.03 ± 0.02	0.01 ± 0.01	1.81 (1.50; 2.19)	***	2.33 (1.93; 2.81)	***	0.82 (0.68; 0.99)	*

The effect of acute exercise on immune cell concentrations is presented as raw concentration data and as relative change from resting pre-exercise (baseline) values using linear mixed models. Linear mixed models are based on log transformed data and results are presented as EMDs and should be interpreted as follows: an EMD of 1.3 is equivalent to a relative increase of 30% from i.e., baseline to the watt-max test. A main effect of time was found for all listed outcomes (p < 0.001). Monocytes and neutrophils were assessed using the Sysmex XN cell counter. T cells (CD3^+^), NKT like cells (CD3^+^CD56^+^), CD8 T cells (CD3^+^CD56^−^CD8^+^), NK cells (CD3^−^ CD14^−^ CD16^+^ CD56^dim^; CD3^−^ CD14^−^ CD16 ± CD56^bright^) were identified using separate flow cytometry experiments. Data on total lymphocytes is published elsewhere (Djurhuus et al., 2022a). *Abbreviations: CI (95% Confidence interval), EMD (estimated mean difference), p (p-value); NK (natural killer), ns (not significant*; *p* > 0.05); ***p < 0.001; **p < 0.01; *p < 0.05.

**Fig. 1:**
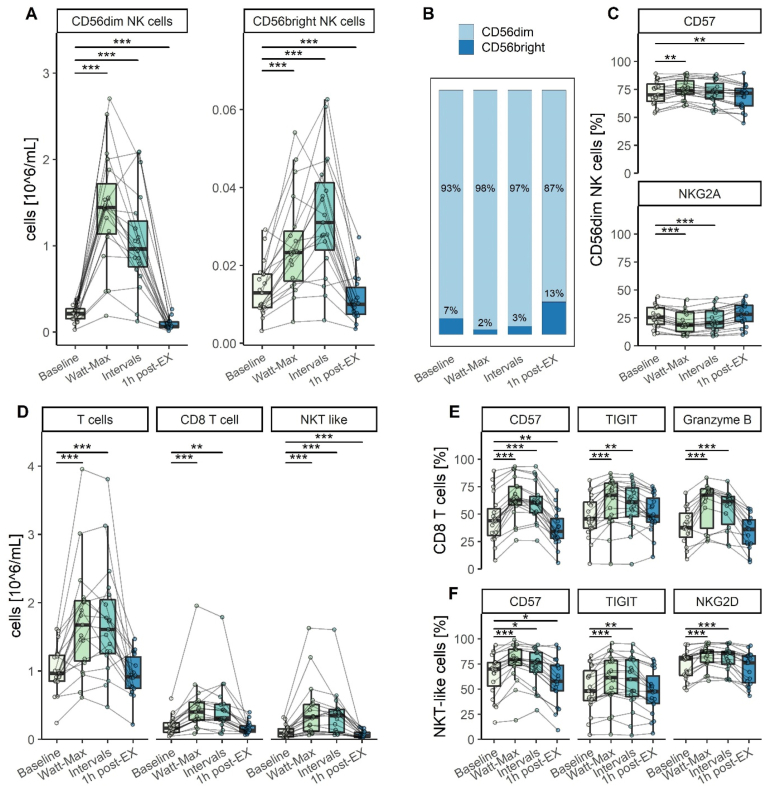
**T and NK cell response during acute exercise.** The NK cell response to acute exercise divided into CD3^−^ CD14^−^ CD16^+^ CD56^dim^ and CD3^−^ CD14^−^ CD16^+/-^ CD56^bright^ NK cells (A). Distribution of NK cell subsets presented as mean percent (B). Selected surface marker expression of CD56^dim^ NK cells (C). The T cell response is divided into all T cells (CD3^+^), CD8 T cells (CD3^+^CD56^−^CD8^+^) and NKT-like cells (CD3^+^CD56^+^) (D). Selected surface marker expression of CD8 T cells (E) and NKT-like cells (F) during acute exercise. For all plots: n = 19 except (CD56^dim^ NKG2A: n = 18 and CD8 T cells Granzyme B: n = 14) ***p < 0.001; **p < 0.01; *p < 0.05.

NK cell subsets were defined as CD3^−^ CD14^−^ CD16^+/-^ CD56^bright^ or CD3^−^ CD14^−^ CD16^+^ CD56^dim^ expressing lymphocytes. Exercise mobilized NK cells to the circulation and NK cells dropped below baseline values 1-h post exercise (Fig. 1A; Table 2) with a preferential mobilization and egress of CD56^dim^ (Fig. 1B). Surface marker expression of CD56^dim^ NK cells revealed a slight increase of CD57 and CD226, whereas NKG2A and NKG2C decreased with exercise (Fig. 1C and Supplementary Table 2). For CD56^bright^ NK cells, minor increases of Granzyme-B, Perforin, and TIGIT were observed with exercise (Supplementary Table 2). Exercise did not change ADRB2, CD16, CD96, NKG2D, CD8, Nkp30, Nkp44, and Nkp46 (Supplementary Table 2). One-hour post-exercise, CD56^dim^ NK cells exhibited slightly lower levels of CD57 and CD8 while CD56^bright^ NK cells showed lower levels of ADRB2, Granzyme-B, and TIGIT (Fig. 1C and Supplementary Table 2).

T cell subsets were defined as CD8 positive T cells (CD3^+^ CD56^−^ CD8^+^) and NKT-like cells (CD3^+^ CD56^+^). Exercise led to a significant mobilization in overall T (CD3^+^), CD8 T, and NKT-like cells with NKT-like cells showing the most prominent increase. During the recovery at 1-h post-exercise, NKT-like cells dropped below baseline values (Fig. 1D; Table 2). During exercise, surface marker expression of CD8 T cells exhibited elevated levels of CD57, TIGIT, granzyme-B, NKG2C, CD16, and Perforin while CD96 and CD226 were decreased. Similarly, NKT-like cells revealed an increase in CD57, TIGIT, NKG2C, NKG2D, CD8, Granzyme-B, and Perforin while CD96 decreased with exercise. One-hour post exercise, CD8 T cells revealed a lower expression of CD57, NKG2C, and Perforin compared to baseline levels. Similarly, CD57 decreased below baseline levels for NKT-like cells (Fig. 1F and Supplementary Table 3).

### 3.2 NK cell cytotoxic activity during and after acute exercise

**Fig. 2:**
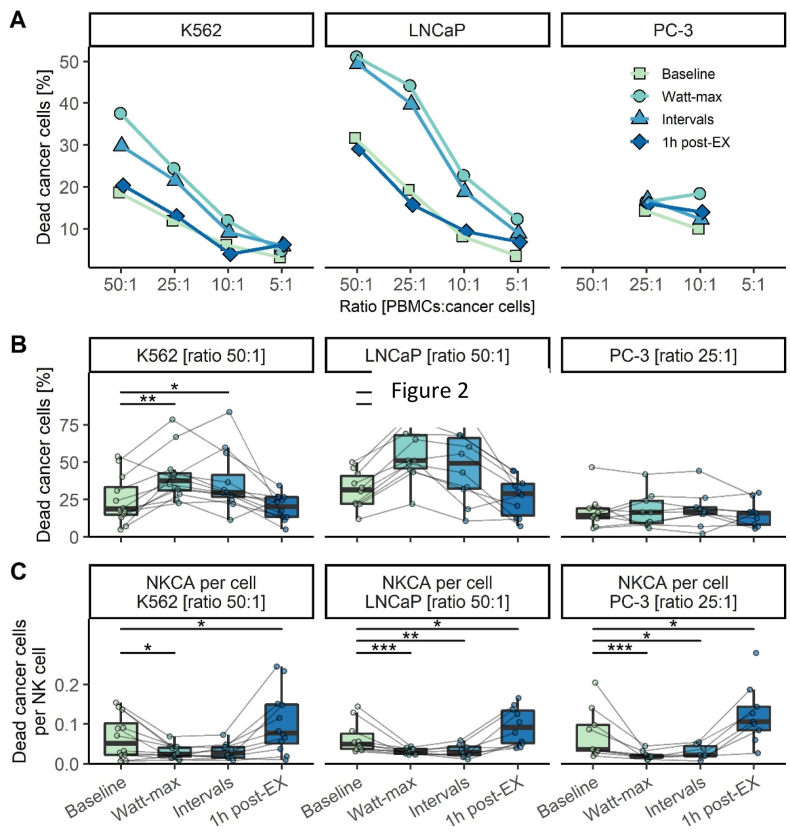
**NK cell cytotoxic activity (NKCA) during exercise.** NKCA presented as mean [%] of dead cancer cells across all ratios (50:1 to 5:1) (A). Individual NKCA at 50:1 or 25:1 ratio (B). NKCA per-cell at 50:1 or 25:1 ratio (C). Replicates: K562 (n = 12); LNCap (n = 10); PC-3 (n = 9). Abbreviations: NKCA (natural killer cell cytotoxic activity). ***p < 0.001; **p < 0.01; *p < 0.05.

We investigated the effect of acute exercise on anti-cancer immunity using an NK cell cytotoxicity assay for a subset of patients (K562: n = 12; LNCaP: n = 10; PC-3: n = 9) ranging from ratios of 50:1 to 5:1 (Fig. 2A). Individual changes displayed a uniform and significant increase of NK cytotoxic activity (NKCA) with exercise against the cell lines K562 and LNCaP but not PC-3 (Fig. 2B; Supplementary Table 4). The NKCA per-cell analyses, based on absolute NK cells present in the assay, showed a significant decrease with acute exercise compared to baseline against the cell lines LNCaP, PC-3, and K562 at the 50:1 ratio (Fig. 2C). For lower ratios, trends were found for a lower NKCA per-cell during exercise (Supplementary Table 4). No change was observed for NKCA during the recovery period, but NKCA per-cell increased during the recovery at 1-h post exercise compared to baseline for the cell lines LNCaP, PC-3 and K562 at the 50:1 ratio (Fig. 2C and Supplementary Table 4).

### 3.3 The effect of baseline inflammation on the exercise-mediated change in immune function

We evaluated the influence of inflammatory signals in the acute exercise response via the association of inflammatory markers (IL-6, TNF-α and CRP) at baseline with immune-related outcomes during acute exercise. IL-6 and CRP correlated with lymphocyte and monocyte concentration at baseline, and IL-6 inversely correlated with the fold-change of lymphocytes during the watt-max test (Table 3).

**Table 3:** Correlations of baseline inflammation and cell populations.

	Baseline concentration						Fold change increase from baseline to the watt-max test					
	Baseline IL-6		Baseline CRP		Baseline TNF-α		Baseline IL-6		Baseline CRP		Baseline TNF-α	
	R	P-value	R	P-value	R	P-value	R	P-value	R	P-value	R	P-value
Lymphocytes	0.53	0.020	0.45	0.051	0.34	0.156	−0.50	0.029	−0.19	0.430	−0.43	0.063
Neutrophils	0.24	0.300	0.33	0.155	0.21	0.379	−0.18	0.458	−0.03	0.900	−0.31	0.182
Monocytes	0.48	0.032	0.59	0.006	0.43	0.059	−0.11	0.632	0.09	0.711	−0.07	0.776
T cells	0.63	0.004	0.45	0.053	0.38	0.104	−0.38	0.104	−0.25	0.300	−0.15	0.542
CD8 T cells	0.39	0.094	0.28	0.241	0.32	0.185	−0.46	0.050	−0.23	0.340	−0.14	0.567
NKT cells	0.12	0.611	−0.18	0.459	−0.03	0.915	−0.08	0.737	−0.25	0.293	−0.27	0.270
CD56^dim^ NK cells	−0.05	0.842	0.26	0.290	−0.02	0.949	−0.10	0.673	−0.40	0.093	−0.31	0.204
CD56^bright^ NK cells	0.11	0.658	−0.15	0.552	0.06	0.803	0.44	0.059	0.36	0.126	0.18	0.450

Resting pre-exercise (baseline) levels of IL-6, CRP and TNF-α are correlated to baseline cell concentration (cells/μl) and fold change of cell concentrations from baseline to watt-max test; method = Spearman correlation; T cells (CD3^+^), NKT like cells (CD3^+^CD56^+^), CD8 T cells (CD3^+^CD56^−^CD8^+^), NK cells (CD3^−^ CD14^−^ CD16^+^ CD56dim; CD3^−^ CD14^−^ CD16 ± CD56bright). *Abbreviations: NK (natural killer); IL (Interleukin); CRP (C-reactive protein); TNF (tumor necrosis factor)*.

**Fig. 3:**
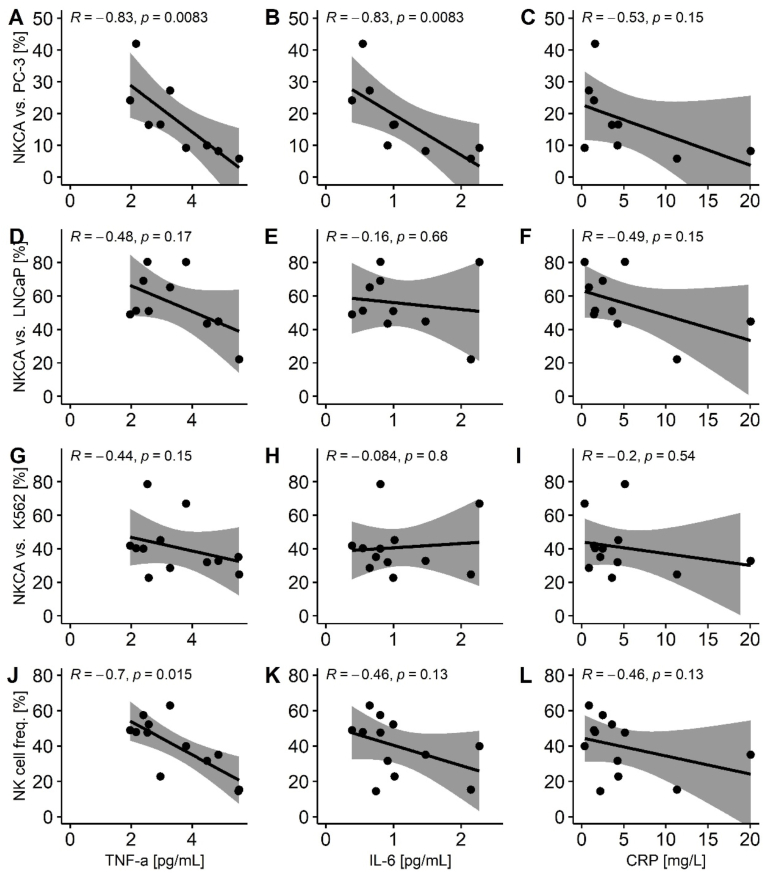
**Correlations of baseline inflammation and** NKCA **after exercise.** Depicted are correlations between baseline levels of IL-6, CRP and TNF-α and NK cell cytotoxic activity after watt-max test vs. PC-3 (A–C), LNCap (D–F), K562 (G–I) at the highest ratio (PBMCs vs. cancer cells) as well as correlations between baseline levels of IL-6, CRP and TNF-α and NK cell proportions (combined CD56^dim^ + CD56^bright^ NK cells) in PBMCs (J–L); method = Spearman correlation; NK cell proportion for participants with available NKCA assay (n = 12), K562 (n = 12); LNCap (n = 10); PC-3 (n = 9). *Abbreviations: NKCA (natural killer cell cytotoxic activity); IL (Interleukin); CRP (C-reactive protein); TNF (tumor necrosis factor).*

Markers of inflammation did not correlate with NK cell concentration, NK cell mobilization during the watt-max test (Table 3) or inhibitory marker expression of NKG2A, TIGIT or CD96 (p > 0.05). Interestingly, baseline inflammation seemed to negatively impact the cytotoxicity against PCa cell lines (Fig. 3). Especially high levels of plasma IL-6 and TNF-α at baseline inversely correlated with the ability to eliminate PC-3 cells during the exercise bout. Furthermore, baseline TNF-α inversely correlated with CD56^dim^ NK cell proportion after the watt-max test for patients with available cytotoxicity data (Fig. 3).

For T cells, IL-6 correlated with an increased baseline concentration of total T cells and inversely correlated with the fold-change of CD8 T cells during the watt-max test (Table 3). Inflammatory markers at baseline did not correlate with inhibitory marker expression of NKG2A, TIGIT or CD96 (p > 0.05).

Independent of exercise-related changes in immune function, inflammatory markers at baseline inversely correlated with the estimated VO_2_ peak of patients obtained during the watt-max test (CRP [R = −0.44; *p* = 0.055]; IL-6 [R = −0.52; *p* = 0.02]; TNF-α [R = −0.55; *p* = 0.014]).

## 4 Discussion

In this exploratory analysis, we performed a detailed characterization of exercise-induced changes in immune cell phenotype and NK cytotoxic activity (NKCA) in patients with early-stage PCa. Our analyses outline three main findings. First, as expected exercise led to an increase in NK, NKT-like and CD8 T cells in the circulation, and especially the mobilized CD8 T cells displayed a pronounced mature and cytotoxic phenotype which was potentially accompanied by cell exhaustion. Secondly, exercise led to an increased NKCA against the cell lines K562 and LNCaP but not PC-3, whereas NKCA per-cell decreased during but improved 1-h post-exercise against all three cell lines compared to baseline. Finally, elevated markers of inflammation at baseline correlated with a lower relative mobilization of immune cells and lower NKCA during exercise.

Immune cell mobilization during acute exercise is a well-described phenomenon in healthy individuals (Pedersen and Hoffman-Goetz, 2000), where acute exercise is known to mobilize cytotoxic NK and T cells to the circulation in an intensity-dependent manner (Campbell et al., 2009). Recently, Hanson and colleagues found increased lymphocyte and NK cell propotion during acute moderate-intensity exercise in patients with PCa with and without androgen deprivation therapy (Hanson et al., 2020). In line with this, we show a pronounced mobilization of both highly differentiated CD56^dim^ and CD56^bright^ NK cells during a strenuous exercise bout. We further expand on these findings showing a similar albeit lesser response of both CD8 T cells and NKT-like cells. Both cell types have been shown to play an important role in anti-cancer immunity (Krijgsman et al., 2018). NKT-like cells combine features of NK and T cells and facilitate the innate and adaptive immune response against cancer (Krijgsman et al., 2018). In addition to classical subset markers, we investigated the impact of acute exercise on a wide array of surface markers. Similar to reports in healthy individuals (Bigley et al., 2014), mobilized CD56^dim^ NK cells in our study displayed a highly differentiated state (CD57^+^ NKG2A^−^ NKG2C^−^). For NKT-like cells, acute exercise led to a more mature and cytotoxic phenotype (CD57^+^ NKG2C^+^ NKG2D^+^ Perforin^bright^ TIGIT^+^). Similar but more pronounced effects were visible for CD8 T cells (CD57^+^ NKG2C^+^ Granzyme-B^+^ Perforin^bright^ TIGIT^+^). Interestingly, exercise led to a preferential mobilization of NKT-like and CD8 T cells harboring the inhibitory receptor TIGIT, which is associated with T cell exhaustion (Blake et al., 2016) and could support the previously seen selective mobilization of senescent T cells during acute exercise (Simpson et al., 2008). Rodent and *in vitro* cancer models showed that targeting the immune checkpoint molecule TIGIT might restore T cell function (Blake et al., 2016). Thus, exercise in combination with immunotherapy targeting TIGIT may result in therapeutic benefits by improving both concentration and function of the T cell mediated immune response.

Acute exercise improves NKCA against the K562 leukemia cell line (Zimmer et al., 2017; Rumpf et al., 2021), likely driven by major histocompatibility complex (MHC) recognition of NK cells (Bigley et al., 2014). Here, we show that acute exercise improved NKCA against K562 and LNCaP, but not PC-3, in patients with PCa. LNCaP cells possess a prostate cell-like phenotype, represent the majority of clinical cases (Tai et al., 2011), and are partly MHC deficient (Sanda et al., 1995), whereas PC-3 express MHC molecules (Sanda et al., 1995) and represent a more aggressive clinical behavior (Tai et al., 2011). PC-3 cells are NK cell resistant target cells but NKCA can be improve upon priming or stimulation of NK cells i.e., by IL-2, in patients with PCa (Hood et al., 2019). Hence, the short-term acute exercise or the sampling time applied in this study might be insufficient to show an improvement in NKCA against NK cell resistant cell lines. Here, the long-term exposure to physical activity involving T cell responses (Emery et al., 2022) might provide a platform to promote anti-cancer effects of exercise towards late-stage cancer. Similar to K562, LNCaP is an NK cell sensitive cell line, and the observed increase of absolute NK cells during exercise likely led to the observed improvements in NKCA. Whether the observed effect of acute exercise on NKCA in the circulation translates to cancerous prostate tissue has not been investigated. In a recent study we found no difference in NK cell infiltration into tumor tissue in patients with early-stage prostate cancer randomized to exercise or control (Djurhuus et al., 2022a, 2022b). However, we found a positive correlation between the amount of high-intensity training sessions and NK cell infiltration into the tumor tissue indicating a potential dose-dependent relationship (Djurhuus et al., 2022b).

Interestingly, NKCA per-cell decreased with exercise and improved during the 1-h recovery period post-exercise against the cell lines K562, LNCap, and PC-3. This is in contrast with a previous report proposing a selective effect of exercise on NKCA per-cell in target cell lines expressing the MHC complex (Bigley et al., 2014). However, earlier reports showed that during the recovery period after high- but not moderate-intensity exercise, NKCA per-cell increased against K562 (Nieman et al., 1993), highlighting a potential role of exercise intensity. Improvements in NKCA per-cell during the recovery period could describe a biological response towards the redistribution of NK cells following exercise. Despite a markedly reduced NK cell concentration in the bloodstream during recovery, NKCA did not fall below values at baseline in this study, potentially mediated by the observed improved NKCA per-cell. A recent meta-analysis in healthy individuals concluded that NKCA does not decline during the recovery phase after exercise compared to non-exercising controls (Rumpf et al., 2021). Hence, our findings support the dogma of an improved immune function during the recovery period of acute exercise (Campbell and Turner, 2018). Speculatively, the decrease of NKCA per-cell during exercise could describe the other side of this biological response towards NK cell redistribution. The several-fold increased concentration of NK cells could trigger feedback mechanisms to minimize e.g., potential damage resulting from off-target activation. Factors changing NKCA per-cell and thereby NK cell function with exercise have not yet been fully identified. Further studies utilizing an unbiased omics or sequencing approach are needed to identify underlying mechanisms.

A potential interaction between systemic inflammation and the acute exercise-dependent immune cell profile may be an important mediating factor in subjects’ individual responses. NKCA is predominantly driven by the increase in NK cell concentration during exercise (Zimmer et al., 2017) but might be modifiable by signaling pathways. To date, the involvement of stress hormones and cytokines in NKCA is not well understood, which is in part due to methodological discrepancies between *in-vitro* and *in-vivo* approaches (Gotlieb et al., 2015). Still, TNF-α secreted by NK cells plays an important role in NKCA (Wang et al., 2012), and serum obtained 1-h post-exercise from healthy individuals can increase NKCA (Gupta et al., 2018). Hence, short-term exposure to cytokines may improve NKCA, whereas chronic stimulation of immune cells by e.g., inflammatory signals might lead to immune-cell exhaustion (Wherry and Kurachi, 2015), subsequently decreasing NKCA. In exploratory analyses, we evaluated the impact of inflammatory markers at baseline on immune cell concentrations and NKCA during acute exercise. We found that the monocyte, overall lymphocyte, and T cell concentration at baseline correlated with plasma IL-6 at baseline. IL-6 promotes survival of both T cells and monocytes (Lotz et al., 1988; Roca et al., 2009) and may therefore lead to accumulation or recruitment of immune cells to the circulation. In addition, baseline plasma IL-6 inversely correlated with the fold-change increase of overall lymphocytes and CD8 T cells, potentially driven by the increased number of immune cells present in circulation already pre-exercise. Further, both plasma IL-6 and TNF-α at baseline inversely correlated with NKCA after an all-out watt-max test, likely driven by a lower NK cell proportion. However, we did not find a correlation between markers of inflammation and inhibitory cell surface markers e.g., TIGIT, in an attempt to quantify cell exhaustion. Future studies should investigate the relationship between low-grade inflammation and other T and NK cell exhaustion markers such as PD-1, Tim-3 and IFN-γ production (Judge et al., 2020) or plasma IL-10 and IFN-γ (Wherry and Kurachi, 2015) during exercise. Taken together, although speculative, these results indicate an interference of low-grade inflammation at baseline in the exercise-mediated improvement of NKCA. Our results may support and extend the cancer immunogram (Blank et al., 2016), a framework to visualize the interaction between the immune system and the state of cancer, showing that inflammatory signals may subdue anti-cancer responses even during acute exercise.

### 4.1 Limitations

This present study has acknowledgeable limitations. Patients were relatively fit but represented the general clinical average of patients offered curatively intended surgery. Furthermore, due to late experimental implementation, we could only investigate NKCA in a subset of participants. While the calcein-based assay correlates well with the predominately older studies that applied ^51^Cr-release assay (Neri et al., 2001), other methods e.g., a flow cytometry-based NKCA assay would provide a more sensitive and more flexible approach to investigate underlying mechanisms. Finally, studies have highlighted the interplay of latent viral infections such as Cytomegalovirus in the exercise response (Turner et al., 2010), a factor inaccessible in this study.

### 4.2 Conclusion & clinical perspective

In summary, acute exercise resulted in a selective mobilization of cytotoxic immune cells and improved NKCA against target cell lines K562 and LNCaP in patients with early-stage PCa. Elevated inflammation might impair the exercise-mediated mobilization as well as the exercise-improved NKCA, highlighting the need for more individualized frameworks such as the cancer immunogram (Blank et al., 2016) to characterize the interactions between the immune system and the cancer environment in clinical exercise studies. Therapies to lower the inflammatory burden might therefore improve the anti-cancer effect of acute exercise. These findings comprise hypothesis-generating work and suggest that well-designed exercise trials should include immune cell parameters and frameworks as well as clinically relevant long-term outcomes such as biochemical progression and/or treatment response in their design.

## Role of the funding source

This study was supported by grants from the 10.13039/501100003554Lundbeck Foundation. The Centre for Physical Activity Research (CFAS) is supported by 10.13039/501100007437TrygFonden (ID 101390 and ID, 20045). The funding source(s) had no involvement in study design; in the collection, analysis and interpretation of data; in the writing of the report; and in the decision to submit the article for publication.

## Declaration of competing interest

The authors declare that they have no known competing financial interests or personal relationships that could have appeared to influence the work reported in this paper.

## Data Availability

Data will be made available on request.
