# Supplementary material for: The effects of acute exercise and inflammation on immune function in early-stage prostate cancer
Source: Brain Behav Immun Health. 2022 Sep 7;25:100508. doi: 10.1016/j.bbih.2022.100508 (PMC9483738; doi:10.1016/j.bbih.2022.100508)
Supplement: Multimedia component 1 [file mmc1.docx]

SUPPLEMENT:


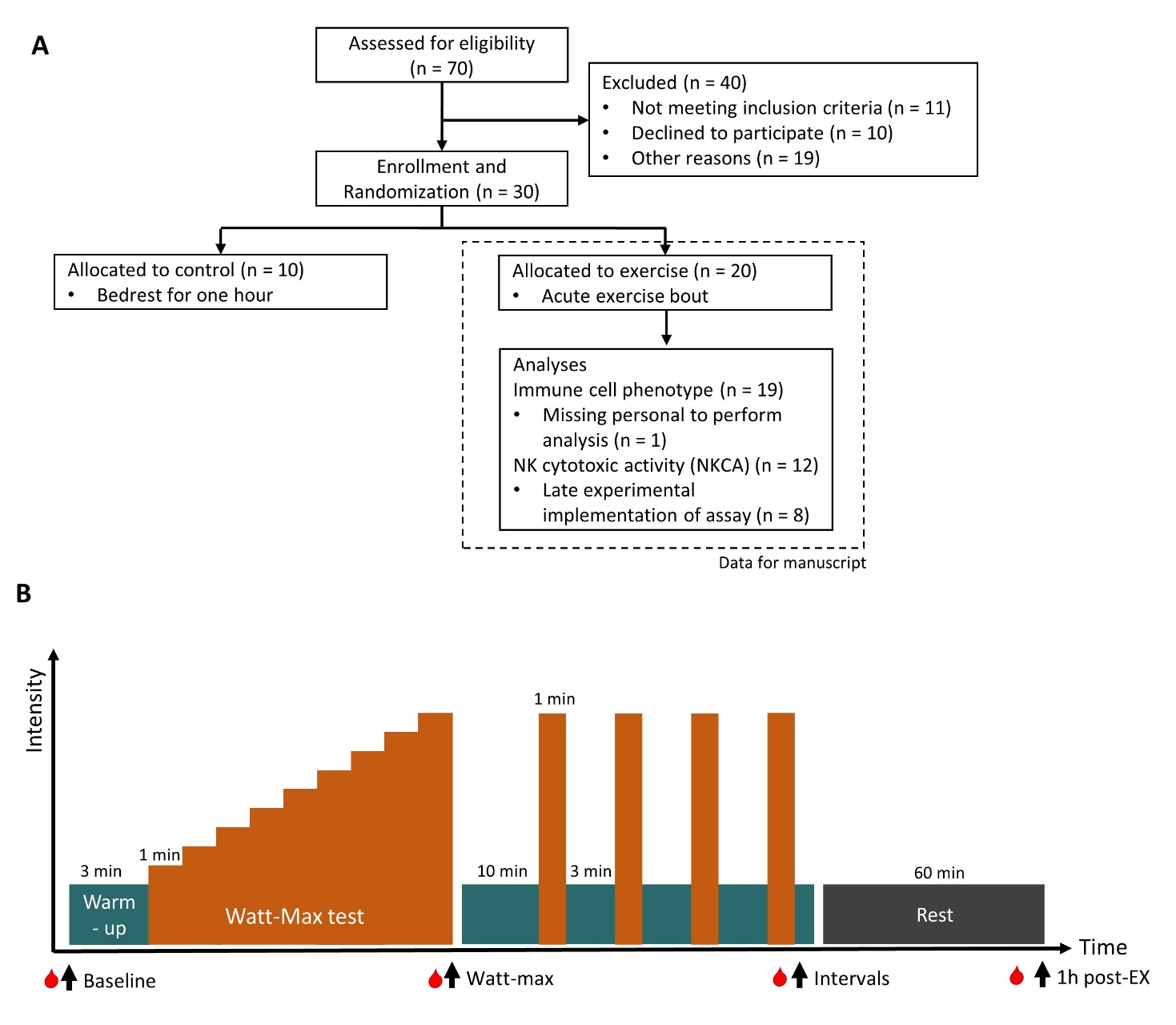


Supplementary Figure 1: Study design. (A) Patient flow of the primary analysis (Clinical Trials identifier: NCT03675529) (Djurhuus et al., 2022) and overview of data included for this manuscript (highlighted with dashed box). This secondary analysis investigated the impact of one acute bout of exercise on immune function in patients with early-stage prostate cancer. All 20 patients completed the acute exercise session and provided blood samples for analyses. Differential count, which includes monocyte, neutrophil and lymphocyte cell count, obtained from the Department of Clinical Biochemistry at Rigshospitalet were obtained from 20/20 patients. The immune cell profiling, which includes the flow cytometric assessment of lymphocyte subpopulation, was performed for 19/20 patients (in unique cases, analysis of the expression of receptor surface markers as percentage was not possible due to insufficient separation of the positive and negative population; for details see supplementary table 2 and 3). The analysis of NK cytotoxic activity (NKCA) was implemented during the ongoing trial. Therefore, data is only available from patients in 12/20 consecutive cases for K562 and in 10/20 consecutive cases for LNCap as well as PC-3. Finally, in one case not enough PBMCs were available to achieve the 25:1 condition for NKCA against the PC-3 cell line.
 Inclusion criteria: men with histologically verified localized prostate adenocarcinoma undergoing curative intended radical prostatectomy. Patients were identified by urologic physicians and screened for study-eligibility via telephone. Exclusion criteria included: age <18 years, other malignancy requiring active treatment, Eastern Cooperative Oncology Group (ECOG) or World Health Organization (WHO) performance status >1, current treatment with beta-blockers, physical disabilities contradicting exercise, allergy to pimonidazole or the inability to read and understand Danish. All participants gave informed consent before undergoing any procedures. The study was preregistered at www.clinicaltrials.gov (NCT03675529).
 (B) Graphical representation of study design and sampling timepoints. In total, four blood samples were obtained at resting pre-exercise (baseline) conditions, immediately after the watt-max test, immediately after the last high-intensity interval and one-hour post-exercise. The watt-max test was initiated with a 3 min warm-up at 70 watts followed by an incremental increase of 20 watts every minute until exhaustion. Immediately after the watt-max test, patients performed 10 minutes of light pedaling at 30% of watt-max to recover. Next, patients performed four one-minute high-intensity intervals at 100% of watt-max, interspersed by three minutes of recovery at 30% of watt-max.

*References:*

Djurhuus SS, Schauer T, Simonsen C, Toft BG, Jensen ARD, Erler JT, et al. Effects of Acute Exercise Training on Tumor Outcomes in Men with Localized Prostate Cancer: A Randomized Controlled Trial. Physiol Rep 2022; in press.


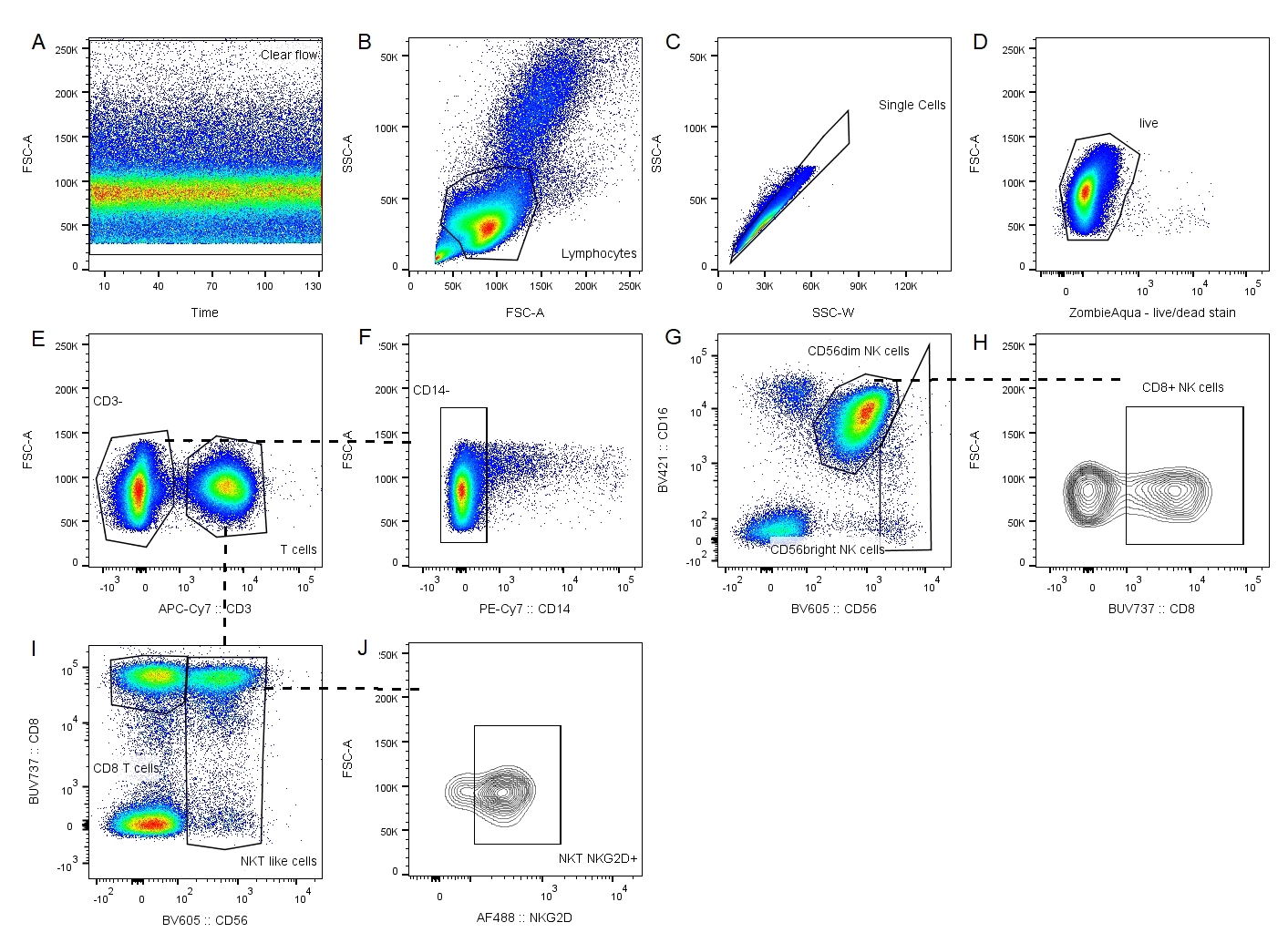


Supplementary Figure 2: Gating strategy. Graphical representation of the gating strategy used to identify lymphocyte subpopulation i.e., NK cells (CD3- CD14- CD16+ CD56^dim^; CD3- CD14- CD16+/- CD56^bright^), NKT-like cells (CD3+ CD56+) and CD8 T cells (CD3+ CD56- CD8+). Gates (A) to (G) are identical between all staining panels (supplementary table 1). Gate (I) was similar between all panels with the exception of the CD8 conjugate. Gates (H) and (J) represent examples on how populations were defined depending on functional markers e.g., NKG2D and were different between the staining panels (supplementary table 1). (A) Selection of clear flow using FSC signal stability across the sample run; (B) selection of lymphocyte population using FSC and SSC parameters; (C) selection of single cells; (D) selection of live cells by gating cells negative for the Zombie Aqua dye; (E) selection of overall T cells (CD3+) and CD3- cells; (F) selection of CD3- CD14- cells; (G) final selection gate for CD56^dim^ (CD3- CD14- CD56^dim^) and CD56^bright^ NK cells (CD3- CD14- CD56^bright^); (H) exemplary gating of CD8 on CD56^dim^ NK cells (gating supported by FMO controls); (I) Selection of T cell subpopulations i.e., CD8 T cells (CD3+ CD56- CD8+) and NKT-like cells (CD3+ CD56+); (J) exemplary analysis of NKG2D on NKT-like cells (gating supported by FMO controls).
 All lymphocyte subpopulations were expressed as proportions [%] of live and single-cell lymphocytes (D). In combination with the absolute lymphocyte concentration from the Department of Clinical Biochemistry acquired on a Sysmex XN automated cell counter, the concentration of lymphocyte subpopulations was calculated. All receptor expression profiles (supplementary figure 2 & 3) were acquired from gates similar to (H) and (J) and expressed as either proportions [%] or median fluorescence intensities (mfi) of the respective lymphocyte subpopulation. *Abbreviations: NK (natural killer), FSC (forward scatter), SSC (side scatter), FMO (Fluorescence minus one).*

Supplementary table 1: FACS staining material overview

| **Name** | | **Fluorochrome** | **Clone** | **Vendor** |
| --- | --- | --- | --- | --- |
| Human BD Fc Block™ | | - | - | BD |
| Live-dead staining (Zombie Aqua) | | - | - | BioLegend |
| Permeabilization buffer | | - | - | R&D systems |
| Brilliant stain buffer | | - | - | BD |
| FACS buffer  (PBS + 2% FBS) | 1x PBS (pH 7.4) | - | - | gibco |
|  | Fetal Bovine Serum | - | - | gibco |
| Total Antibody Compensation Bead Kit | | - | - | ThermoFisher |
| CompBeads | | - | - | BD |
| **Base colors included in every panel** | | | | |
| CD3 | | APC-H7 | SK7 | BD |
| CD16 | | BV421 | 3G8 | BD |
| CD14 | | PE-Cy7 | MφP9 | BD |
| CD56 | | BV605 | NCAM16.2 | BD |
| **Panel 1** | | | | |
| CD8 | | BUV737 | SK1 | BD |
| CD314 – (NKG2D) | | Alexa-Fluor 488 | #149810 | R&D systems |
| CD337 – (Nkp30) | | BV786 | p30-15 | BD |
| CD335 – (Nkp46) | | BV711 | 9E2/Nkp46 | BD |
| CD336 – (Nkp44) | | APC | P44-8 | BioLegend |
| TIGIT – (VSTM3) | | PE | A15153G | BioLegend |
| **Panel 2** | | | | |
| CD8 | | BUV737 | SK1 | BD |
| CD159a – (NKG2A) | | Alexa-Fluor 647 | #131411 | R&D systems |
| CD159c – (NKG2C) | | PE | #134591 | R&D systems |
| ADRB2 | | Alexa-Fluor 488 | *polyclonal* | Bioss Inc |
| CD96 | | BV711 | 6F9 | BD |
| **Panel 3** | | | | |
| CD8 | | FITC | HIT8α | BD |
| Perforin* | | BV711 | dG9 | BioLegend |
| Granzyme B* | | PE | GB11 | BD |
| CD57 | | PE-Cy5 | NK-1 | Abeomics |
| CD226 – (DNAM.1) | | BUV395 | DX11 | BD |

*Perforin and granzyme B represent intracellular targets

**Supplementary table 2: NK cell surface marker response during acute exercise**

|  |  |  | Raw data | | | |  | Linear mixed model statistics | | | | | |
| --- | --- | --- | --- | --- | --- | --- | --- | --- | --- | --- | --- | --- | --- |
|  |  |  | Mean ± SD | | | | Main effect  of time | Baseline vs. watt-max test | | Baseline vs. intervals | | Baseline vs. 1h post-EX | |
| variable | n | Unit | baseline | post watt-max | post-intervals | 1h post-EX | p-value | EMD (CI) | p-value | EMD (CI) | p-value | EMD (CI) | p-value |
| **Surface markers of CD3- CD14- CD16+ CD56^dim^ NK cells** | | | | | | | | | | | | | |
| *ADRB2 | 14 | mfi | 690 ± 354 | 697 ± 372 | 679 ± 357 | 643 ± 355 | 0.020 | 1.01 (0.93; 1.09) | 0.997 | 0.98 (0.91; 1.06) | 0.905 | 0.92 (0.85; 0.99) | 0.024 |
| CD226 | 12 | % | 83.1 ± 11.7 | 93.7 ± 6.4 | 88.3 ± 8.5 | 80.9 ± 15.2 | <0.001 | 1.14 (1.03; 1.25) | 0.009 | 1.07 (0.97; 1.18) | 0.294 | 0.97 (0.87; 1.07) | 0.773 |
| CD57 | 19 | % | 71.7 ± 10.7 | 75.3 ± 9.1 | 72.1 ± 10.6 | 68.3 ± 11.3 | <0.001 | 1.05 (1.02; 1.09) | 0.002 | 1.01 (0.97; 1.04) | 0.966 | 0.95 (0.92; 0.98) | 0.002 |
| CD8 | 19 | % | 38.6 ± 14.3 | 38.6 ± 14.8 | 37.4 ± 13.8 | 36.7 ± 14 | 0.010 | 1 (0.95; 1.04) | 0.995 | 0.97 (0.93; 1.01) | 0.161 | 0.95 (0.91; 0.99) | 0.013 |
| *CD96 | 19 | mfi | 135 ± 33.5 | 122 ± 28.2 | 122 ± 29.4 | 130 ± 31.6 | <0.001 | 0.91 (0.87; 0.94) | <0.001 | 0.91 (0.87; 0.95) | <0.001 | 0.97 (0.93; 1.01) | 0.169 |
| Granzyme-B | 19 | mfi | 2780 ± 3938 | 2407 ± 2520 | 2398 ± 3399 | 2959 ± 4372 | 0.170 |  |  |  |  |  |  |
| NKG2A | 18 | % | 25.9 ± 9.7 | 21 ± 9.8 | 23 ± 10 | 27.8 ± 10.4 | <0.001 | 0.78 (0.73; 0.84) | <0.001 | 0.87 (0.81; 0.93) | <0.001 | 1.07 (0.99; 1.15) | 0.105 |
| NKG2C | 18 | % | 18.6 ± 20.4 | 16.7 ± 21 | 17.6 ± 20.6 | 19 ± 18.5 | <0.001 | 0.8 (0.72; 0.88) | <0.001 | 0.88 (0.8; 0.97) | 0.005 | 1.07 (0.98; 1.18) | 0.204 |
| *NKG2D | 19 | mfi | 222 ± 45.5 | 203 ± 41.8 | 209 ± 41.3 | 225 ± 44.7 | <0.001 | 0.91 (0.88; 0.95) | <0.001 | 0.94 (0.91; 0.98) | 0.002 | 1.02 (0.98; 1.06) | 0.734 |
| NKP30 | 19 | mfi | 502 ± 232 | 505 ± 232 | 487 ± 227 | 491 ± 226 | 0.390 |  |  |  |  |  |  |
| NKP44 | 19 | mfi | 23.8 ± 11.3 | 22.2 ± 12.4 | 21.4 ± 11.3 | 24.1 ± 10.4 | 0.010 | 0.91 (0.81; 1.03) | 0.205 | 0.89 (0.79; 1.01) | 0.067 | 1.04 (0.92; 1.17) | 0.864 |
| NKP46 | 16 | % | 92.5 ± 4.4 | 93.5 ± 3.5 | 92.8 ± 4.1 | 92.2 ± 4.5 | 0.020 | 1.01 (1; 1.02) | 0.061 | 1 (0.99; 1.02) | 0.792 | 1 (0.99; 1.01) | 0.899 |
| *Perforin | 19 | mfi | 2221 ± 2530 | 2298 ± 2378 | 2297 ± 3218 | 2048 ± 2282 | 0.030 | 1.08 (0.94; 1.24) | 0.436 | 0.92 (0.8; 1.06) | 0.389 | 0.94 (0.82; 1.07) | 0.564 |
| TIGIT | 19 | % | 84.4 ± 9.8 | 83.8 ± 10 | 83.9 ± 10 | 84.7 ± 9.5 | 0.030 | 0.99 (0.98; 1) | 0.314 | 0.99 (0.98; 1) | 0.406 | 1 (0.99; 1.02) | 0.662 |
| **Surface markers of CD3- CD14- CD16+/- CD56^bright^ NK cells** | | | | | | | | | | | | | |
| ADRB2 | 14 | mfi | 219 ± 201 | 223 ± 249 | 162 ± 76 | 124 ± 27 | 0.001 | 1 (0.77; 1.28) | 1.000 | 0.87 (0.67; 1.12) | 0.418 | 0.7 (0.54; 0.89) | 0.003 |
| CD226 | 7 | % | 80.4 ± 18.9 | 90.9 ± 15.6 | 84.1 ± 19 | 83.9 ± 20.6 | 0.380 |  |  |  |  |  |  |
| CD8 | 16 | % | 32.4 ± 11.5 | 33 ± 10.8 | 32 ± 9.9 | 30.6 ± 10.6 | 0.060 |  |  |  |  |  |  |
| *CD96 | 19 | mfi | 390 ± 101 | 413 ± 106 | 392 ± 104 | 358 ± 101 | <0.001 | 1.06 (1.01; 1.11) | 0.021 | 1 (0.95; 1.06) | 0.996 | 0.91 (0.87; 0.96) | <0.001 |
| Granzyme-B | 19 | mfi | 987 ± 1643 | 1167 ± 1534 | 1284 ± 2059 | 816 ± 1263 | <0.001 | 1.42 (1.22; 1.66) | <0.001 | 1.28 (1.1; 1.5) | <0.001 | 0.83 (0.71; 0.97) | 0.013 |
| NKG2A | 19 | % | 79.6 ± 10.5 | 78.2 ± 6.7 | 79.3 ± 6.3 | 79.2 ± 8.7 | 0.910 |  |  |  |  |  |  |
| NKG2D | 19 | % | 85.3 ± 8.9 | 86 ± 7.6 | 87.2 ± 7.3 | 85.2 ± 8.8 | 0.010 | 1.01 (0.99; 1.03) | 0.571 | 1.02 (1; 1.04) | 0.016 | 1 (0.98; 1.02) | 1.000 |
| *NKP30 | 19 | mfi | 532 ± 194 | 583 ± 196 | 561 ± 182 | 478 ± 174 | <0.001 | 1.1 (1.03; 1.18) | 0.002 | 1.06 (0.99; 1.13) | 0.096 | 0.9 (0.84; 0.96) | <0.001 |
| *NKP44 | 19 | mfi | 56.6 ± 19.4 | 51.3 ± 18.3 | 54.8 ± 20.2 | 59.3 ± 17.7 | <0.001 | 0.91 (0.84; 0.98) | 0.011 | 0.96 (0.89; 1.04) | 0.598 | 1.07 (0.98; 1.15) | 0.165 |
| NKP46 | 19 | % | 92 ± 4.2 | 90.4 ± 5.5 | 92.6 ± 4 | 92.2 ± 3.5 | 0.030 | 0.98 (0.96; 1) | 0.112 | 1.01 (0.99; 1.03) | 0.845 | 1 (0.98; 1.02) | 0.989 |
| Perforin | 19 | mfi | 627 ± 707 | 977 ± 1063 | 983 ± 1505 | 512 ± 530 | <0.001 | 1.47 (1.27; 1.71) | <0.001 | 1.22 (1.05; 1.41) | 0.005 | 0.86 (0.74; 1) | 0.050 |
| TIGIT | 19 | % | 26.6 ± 7.5 | 35.4 ± 9.8 | 32.5 ± 9.8 | 22.9 ± 8.3 | <0.001 | 1.33 (1.2; 1.47) | <0.001 | 1.22 (1.1; 1.34) | <0.001 | 0.84 (0.76; 0.93) | <0.001 |

Estimates obtained from linear mixed models; data is presented as estimated mean difference and should be interpreted as follows: an EMD of 1.10 is equivalent to an increase of 10% from i.e., resting pre-exercise (baseline) values to the watt-max test. Usually, proportions [%] are presented, and MFI is only used if no clear positive population was present. Missing data for proportions is due to unclear separation of positive/negative populations in some samples, except for ADRB2 where antibody solution did not arrive in time. *MFI below 10% relative change, which was determined as biological significant change. *Abbreviations*: *CI (95% Confidence interval), EMD (estimated mean difference); MFI (median fluorescence intensity).*

**Supplementary table 3: T cell surface marker response during acute exercise**

|  |  |  | Raw data | | | |  | Linear mixed model statistics | | | | | |
| --- | --- | --- | --- | --- | --- | --- | --- | --- | --- | --- | --- | --- | --- |
|  |  |  | Mean ± SD | | | | Main effect of time | Baseline vs. watt-max test | | Baseline vs. intervals | | Baseline vs. 1h post-EX | |
| Target | n | Unit | baseline | post watt-max | post-intervals | 1h post-EX | p-value | EMD (CI) | p-value | EMD (CI) | p-value | EMD (CI) | p-value |
| **Surface markers of CD3+ CD56- CD8+ T cells** | | | | | | | | | | | | | |
| ADRB | 14 | mfi | 120 ± 26.3 | 111 ± 15.7 | 116 ± 15.6 | 131 ± 59 | 0.510 |  |  |  |  |  |  |
| CD16 | 19 | mfi | 68.8 ± 26.3 | 82.8 ± 44 | 80 ± 31 | 61.3 ± 22.1 | <0.001 | 1.16 (1; 1.34) | 0.041 | 1.16 (1.01; 1.34) | 0.036 | 0.89 (0.77; 1.03) | 0.152 |
| CD226 | 16 | % | 79.8 ± 11 | 72 ± 14.7 | 75.5 ± 14.2 | 82.7 ± 10.4 | <0.001 | 0.89 (0.82; 0.96) | 0.002 | 0.94 (0.87; 1.01) | 0.120 | 1.04 (0.96; 1.12) | 0.555 |
| CD57 | 19 | % | 45.8 ± 21.6 | 64.3 ± 18.5 | 61 ± 17.2 | 36.9 ± 16.2 | <0.001 | 1.52 (1.32; 1.76) | <0.001 | 1.45 (1.26; 1.68) | <0.001 | 0.81 (0.7; 0.94) | 0.002 |
| CD96 | 19 | mfi | 499 ± 188 | 375 ± 154 | 382 ± 139 | 540 ± 195 | <0.001 | 0.75 (0.69; 0.83) | <0.001 | 0.78 (0.71; 0.85) | <0.001 | 1.1 (1; 1.2) | 0.051 |
| Granzyme-B | 14 | % | 39 ± 17 | 58.1 ± 20.6 | 54 ± 18.3 | 33.5 ± 15.8 | <0.001 | 1.57 (1.28; 1.93) | <0.001 | 1.44 (1.18; 1.77) | <0.001 | 0.83 (0.68; 1.01) | 0.070 |
| NKG2A | 19 | mfi | 29.4 ± 11.5 | 28.5 ± 14.9 | 27.5 ± 11.3 | 30.9 ± 18.2 | 0.300 |  |  |  |  |  |  |
| NKG2C | 14 | % | 8.3 ± 12 | 13.2 ± 13 | 12.1 ± 12.1 | 8.8 ± 21.2 | <0.001 | 1.91 (1.42; 2.56) | <0.001 | 1.77 (1.32; 2.38) | <0.001 | 0.68 (0.51; 0.91) | 0.007 |
| NKG2D | 19 | % | 93.7 ± 2.1 | 92.2 ± 3 | 92.7 ± 2.8 | 94 ± 2.2 | <0.001 | 0.98 (0.97; 1) | 0.020 | 0.99 (0.98; 1) | 0.209 | 1 (0.99; 1.02) | 0.881 |
| Perforin | 19 | mfi | 448 ± 410 | 874 ± 886 | 889 ± 1321 | 324 ± 244 | <0.001 | 1.71 (1.39; 2.09) | <0.001 | 1.47 (1.2; 1.8) | <0.001 | 0.77 (0.63; 0.94) | 0.008 |
| TIGIT | 19 | % | 47.9 ± 19.2 | 60.6 ± 21.9 | 58.4 ± 20.6 | 50.7 ± 17 | <0.001 | 1.26 (1.1; 1.45) | <0.001 | 1.22 (1.07; 1.41) | 0.002 | 1.12 (0.97; 1.28) | 0.162 |
| **Surface markers of CD3+ CD56+ NKT-like cells** | | | | | | | | | | | | | |
| ADRB | 14 | mfi | 187 ± 82 | 163 ± 46.3 | 175 ± 58 | 192 ± 84 | 0.130 |  |  |  |  |  |  |
| CD16 | 19 | mfi | 187 ± 114 | 321 ± 593 | 195 ± 171 | 360 ± 914 | 0.440 |  |  |  |  |  |  |
| CD226 | 16 | % | 84 ± 11.8 | 79.1 ± 17.3 | 83.2 ± 12.5 | 86.3 ± 8.8 | 0.010 | 0.93 (0.85; 1) | 0.064 | 0.99 (0.91; 1.07) | 0.981 | 1.03 (0.95; 1.12) | 0.689 |
| CD57 | 19 | % | 64.5 ± 21.1 | 76.5 ± 18.7 | 71.8 ± 18.5 | 57.8 ± 22.6 | <0.001 | 1.22 (1.08; 1.37) | <0.001 | 1.15 (1.02; 1.29) | 0.016 | 0.86 (0.77; 0.97) | 0.010 |
| CD8 | 19 | % | 57.9 ± 16.9 | 65.7 ± 16.5 | 63 ± 16.6 | 56 ± 19.1 | <0.001 | 1.15 (1.06; 1.24) | <0.001 | 1.1 (1.01; 1.19) | 0.024 | 0.95 (0.87; 1.03) | 0.274 |
| CD96 | 19 | mfi | 376 ± 172 | 322 ± 137 | 333 ± 150 | 386 ± 161 | <0.001 | 0.87 (0.81; 0.94) | <0.001 | 0.89 (0.82; 0.95) | <0.001 | 1.04 (0.96; 1.12) | 0.558 |
| Granzyme-B | 19 | mfi | 2339 ± 3311 | 2391 ± 2308 | 2223 ± 2743 | 2014 ± 2892 | <0.001 | 1.27 (1.03; 1.56) | 0.022 | 1.02 (0.83; 1.26) | 0.995 | 0.86 (0.7; 1.06) | 0.228 |
| NKG2A | 13 | % | 10.3 ± 9 | 10.3 ± 9.7 | 9.9 ± 8.9 | 10.4 ± 10.2 | 0.650 |  |  |  |  |  |  |
| NKG2C | 16 | % | 20.4 ± 20.6 | 24 ± 22.9 | 23.1 ± 22.9 | 20.2 ± 24.2 | <0.001 | 1.26 (1.02; 1.55) | 0.024 | 1.17 (0.95; 1.44) | 0.182 | 0.84 (0.68; 1.03) | 0.107 |
| NKG2D | 19 | % | 74 ± 13.9 | 82.1 ± 11.4 | 80.8 ± 11.8 | 70.9 ± 15.6 | <0.001 | 1.12 (1.06; 1.18) | <0.001 | 1.1 (1.04; 1.16) | <0.001 | 0.95 (0.9; 1) | 0.080 |
| Perforin | 19 | mfi | 723 ± 649 | 1088 ± 1018 | 1085 ± 1503 | 570 ± 419 | <0.001 | 1.41 (1.2; 1.65) | <0.001 | 1.2 (1.02; 1.41) | 0.019 | 0.85 (0.73; 1) | 0.054 |
| TIGIT | 19 | % | 50.2 ± 22.5 | 59.1 ± 25.1 | 56.9 ± 25.3 | 47.4 ± 21.7 | <0.001 | 1.18 (1.09; 1.29) | <0.001 | 1.11 (1.02; 1.21) | 0.009 | 0.95 (0.88; 1.04) | 0.452 |

Estimates obtained from linear mixed models; data is presented as estimated mean difference and should be interpreted as follows: an EMD of 1.10 is equivalent to an increase of 10% from i.e., resting pre-exercise (baseline) values to the watt-max test. Usually, proportions [%] presented, and MFI is only used if no clear positive population was present. Missing data for proportions is due to unclear separation of positive/negative populations in some samples, except for ADRB2 where antibody solution did not arrive in time. *Abbreviations*: *CI (95% Confidence interval), EMD (estimated mean difference); MFI (median fluorescence intensity).*

Supplementary Table 4: Overview of NKCA and NKCA per-cell across the intervention

|  | |  | Raw data | | | |  | Linear mixed model statistics | | | | | |
| --- | --- | --- | --- | --- | --- | --- | --- | --- | --- | --- | --- | --- | --- |
|  |  |  | Mean ± SD | | | | Main effect of time | Baseline vs. watt-max test | | Baseline vs. intervals | | Baseline vs. 1h post-EX | |
| Cell type | Ratio |  | baseline | post watt-max | post exercise | 1h post-EX | p-value | EMD (95% CI) | p-value | EMD (95% CI) | p-value | EMD (95% CI) | p-value |
| **NK cell cytotoxic activity (NKCA) [%]** | | | | | | | | | | | | | |
| K562 | 50:1 | 12 | 24.6 ± 16.2 | 40.7 ± 16.7 | 36.7 ± 20 | 19.9 ± 8.5 | <0.001 | 16.1 (5.22; 26.98) | 0.002 | 12.09 (1.21; 22.97) | 0.026 | -4.7 (-15.58; 6.18) | 0.635 |
|  | 25:1 | 12 | 14.5 ± 9.6 | 27.3 ± 15.5 | 20.8 ± 11.3 | 12.2 ± 8.7 | <0.001 | 12.83 (5.41; 20.26) | <0.001 | 6.33 (-1.09; 13.76) | 0.114 | -2.26 (-9.68; 5.17) | 0.834 |
|  | 10:1 | 12 | 7.93 ± 7.2 | 14 ± 9.4 | 9.91 ± 6.5 | 6.8 ± 7.6 | 0.020 | 6.1 (0.44; 11.77) | 0.031 | 1.99 (-3.67; 7.65) | 0.766 | -1.13 (-6.79; 4.53) | 0.945 |
|  | 5:1 | 12 | 5.8 ± 7.6 | 7.06 ± 6.5 | 6.55 ± 5.3 | 5.28 ± 6.1 | 0.750 |  |  |  |  |  |  |
| LNCap | 50:1 | 10 | 31.6 ± 12.2 | 55.6 ± 18.2 | 47.6 ± 24.2 | 26.9 ± 13.4 | <0.001 | 24.02 (14.05; 34) | <0.001 | 16.01 (6.04; 25.98) | 0.001 | -4.72 (-14.69; 5.26) | 0.560 |
|  | 25:1 | 10 | 21.9 ± 12.9 | 42.7 ± 18.7 | 38.4 ± 19.6 | 19.5 ± 12.7 | <0.001 | 20.8 (12.2; 29.4) | <0.001 | 16.53 (7.93; 25.13) | <0.001 | -2.32 (-10.92; 6.28) | 0.873 |
|  | 10:1 | 10 | 11.2 ± 12 | 23.1 ± 14.7 | 19.9 ± 15.5 | 13.5 ± 13 | <0.001 | 11.84 (5.74; 17.94) | <0.001 | 8.66 (2.56; 14.77) | 0.004 | 2.31 (-3.79; 8.41) | 0.718 |
|  | 5:1 | 10 | 5.02 ± 8.1 | 11.9 ± 9.5 | 9.78 ± 10.1 | 5.98 ± 8.7 | <0.001 | 6.92 (2.83; 11.01) | <0.001 | 4.76 (0.67; 8.85) | 0.019 | 0.96 (-3.13; 5.05) | 0.912 |
| PC3 | 25:1 | 9 | 17.3 ± 12.2 | 17.7 ± 11.7 | 18.4 ± 12 | 15.5 ± 8.6 | 0.730 |  |  |  |  |  |  |
|  | 10:1 | 11 | 11.7 ± 6.6 | 16.2 ± 12.7 | 15.2 ± 9.4 | 13.9 ± 9.5 | 0.320 |  |  |  |  |  |  |
| **NK cell cytotoxic activity (NKCA) per cell [dead cancer cells per NK cell]** | | | | | | | | | | | | | |
| K562 | 50:1 | 12 | 0.067 ± 0.055 | 0.03 ± 0.017 | 0.032 ± 0.02 | 0.103 ± 0.078 | <0.001 | 0.6 (0.36; 0.98) | 0.041 | 0.69 (0.41; 1.17) | 0.238 | 1.7 (1.03; 2.8) | 0.033 |
|  | 25:1 | 12 | 0.078 ± 0.07 | 0.037 ± 0.023 | 0.038 ± 0.025 | 0.124 ± 0.12 | <0.001 | 0.65 (0.37; 1.15) | 0.181 | 0.64 (0.35; 1.16) | 0.186 | 1.79 (1; 3.21) | 0.049 |
|  | 10:1 | 12 | 0.097 ± 0.088 | 0.046 ± 0.034 | 0.043 ± 0.03 | 0.185 ± 0.25 | 0.010 | 0.57 (0.27; 1.19) | 0.172 | 0.45 (0.21; 0.97) | 0.039 | 1.14 (0.53; 2.43) | 0.964 |
|  | 5:1 | 12 | 0.153 ± 0.18 | 0.044 ± 0.044 | 0.06 ± 0.042 | 0.269 ± 0.39 | 0.010 | 0.44 (0.18; 1.07) | 0.076 | 0.58 (0.23; 1.46) | 0.373 | 1.6 (0.66; 3.91) | 0.465 |
| LNCap | 50:1 | 10 | 0.066 ± 0.041 | 0.031 ± 0.008 | 0.032 ± 0.016 | 0.095 ± 0.048 | <0.001 | 0.54 (0.38; 0.77) | <0.001 | 0.55 (0.37; 0.81) | 0.001 | 1.47 (1.02; 2.11) | 0.034 |
|  | 25:1 | 10 | 0.086 ± 0.065 | 0.047 ± 0.015 | 0.054 ± 0.027 | 0.13 ± 0.076 | <0.001 | 0.7 (0.42; 1.15) | 0.220 | 0.74 (0.43; 1.26) | 0.406 | 1.69 (1.02; 2.79) | 0.039 |
|  | 10:1 | 10 | 0.1 ± 0.14 | 0.06 ± 0.033 | 0.071 ± 0.058 | 0.193 ± 0.19 | 0.680 |  |  |  |  |  |  |
|  | 5:1 | 10 | 0.064 ± 0.2 | 0.056 ± 0.049 | 0.08 ± 0.074 | 0.119 ± 0.31 | <0.001 | 0.66 (0.33; 1.3) | 0.325 | 0.8 (0.39; 1.65) | 0.821 | 2.26 (1.1; 4.65) | 0.023 |
| PC3 | 25:1 | 9 | 0.076 ± 0.063 | 0.021 ± 0.011 | 0.031 ± 0.018 | 0.124 ± 0.074 | <0.001 | 0.35 (0.19; 0.62) | <0.001 | 0.52 (0.28; 0.98) | 0.041 | 1.86 (1.04; 3.32) | 0.033 |
|  | 10:1 | 11 | 0.155 ± 0.15 | 0.054 ± 0.054 | 0.084 ± 0.067 | 0.297 ± 0.26 | 0.001 | 0.25 (0.09; 0.7) | 0.006 | 0.51 (0.17; 1.53) | 0.345 | 1.39 (0.49; 3.9) | 0.810 |

Estimates obtained from linear mixed models; data is shown as absolute changes for NKCA and as estimated mean difference (EMD) for NKCA per cell (after log transformation + back-transformation). EMDs should be interpreted as follows: an EMD of 1.10 is equivalent to an increase of 10% from i.e., resting pre-exercise (baseline) values to the watt-max test. *Abbreviations: CI (95% Confidence interval), EMD (estimated mean difference).*
